# Direct Observation of Reversible Biomolecule Switching Controlled By Electrical Stimulus

**DOI:** 10.1002/admi.201400026

**Published:** 2014-04-04

**Authors:** Alice Pranzetti, Matthew Davis, Chun L Yeung, Jon A Preece, Patrick Koelsch, Paula M Mendes

**Affiliations:** School of Chemical Engineering, University of BirminghamEdgbaston, Birmingham, B15 2TT, UK; School of Chemistry, University of BirminghamEdgbaston, Birmingham, B15 2TT, UK; National ESCA and Surface Analysis Center for Biomedical Problems, Department of Bioengineering, University of WashingtonP.O. Box 351653, Seattle, Washington, 98195–1653, USA

**Keywords:** switchable surfaces, self-assembled monolayers, sum frequency generation, electrochemistry

Control and reversibility of biomolecular interactions at engineered interfaces presents opportunities to develop highly efficient substrates and devices for a wide range of biomedical applications.^1^–^4^ A major challenge nowadays in the field of stimuli-responsive interfaces is to acquire a molecular understanding of the changes occurring at the biointerface upon external stimulation. Herein, we used in situ Sum-Frequency-Generation (SFG) spectroscopy to study changes in molecular orientations in electrically switchable biofunctionalized self-assembled monolayers (SAMs). The bioactivity of a mixed SAM on gold consisting of a biotin-terminated positively charged oligopeptide (biotin-KKKKC) and a tri(ethylene glycol)-terminated thiol is shown to be related to a switch between upward exposure and random orientation of the biotin group in response to positive and negative applied potentials, respectively. The findings reported here support the mechanism by which charged biomolecules control biomolecular interactions, for example, protein binding affinities, and lay the foundation for future studies aiming to explore molecular conformational changes in response to electrical stimuli.

Dynamic surfaces are particularly attractive for biomedical applications and are playing an increasingly important part in the development of highly sensitive biosensors,^5^–^7^ novel drug delivery systems,^8^ and tissue engineering scaffolds.^9^ Stimuli-responsive SAMs have garnered much interest since they can provide a high level of molecular organization and control over the surface properties.^10^ To date, stimuli-responsive SAMs have been able to selectively respond to external inputs such as electrical,^11^–^13^ temperature,^14^,^15^ pH,^16^ and light.^17^,^18^ Switchable SAMs used to control biomolecular interactions via an electrical stimulus are particularly appealing because of their fast response times, ease of creating multiple individually addressable switchable regions on the same surface, as well as low-driven voltage and electric fields that are compatible with biological systems.^19^ Electrically switchable SAMs have been demonstrated to modulate the interactions of surfaces with proteins,^11^–^13^ DNA,^20^,^21^ and mammalian^19^ and bacterial^22^ cells. For instance, an electrically switchable mixed SAM on gold that comprised a positively charged oligopeptide (biotin-KKKKC) and a shorter tri(ethylene glycol)-terminated thiol (TEGT) (**Figure** **1**) was previously^13^ demonstrated by us to be able to control the bioactivity of biotin on the surface and its binding to a specific protein, Neutravidin. High protein binding was observed for an applied positive potential (+0.3 V, bio-active state), while minimal binding was detected for an applied negative potential (-0.4 V, bio-inactive state).

**Figure 1:**
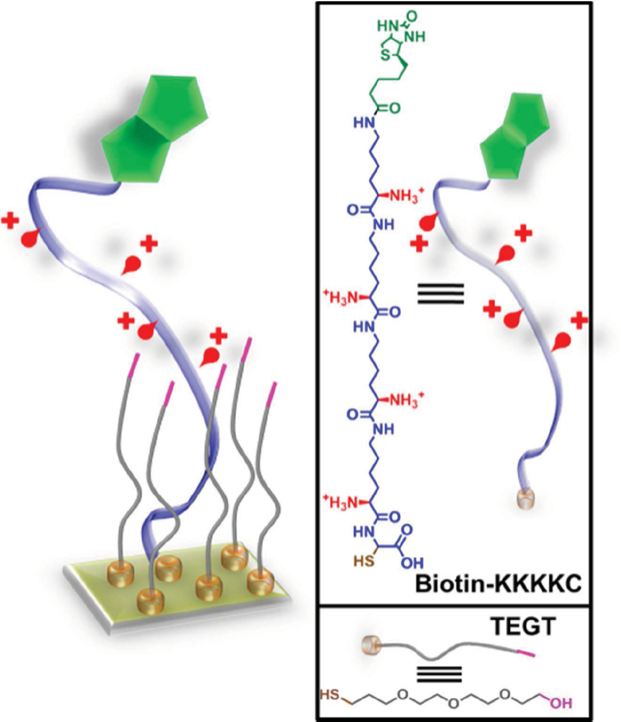
Schematic representation of the biotin-KKKKC:TEGT SAM and chemical structure of the biotin-KKKKC oligopeptide and TEGT.

While the charged molecular backbone or the end group on the structure of the reported electrically switchable SAMs are the hypothesized basis for controlling such biomolecular interactions,^23^ the mechanistic principles underpinning these electrically-driven systems are not fully experimentally proven. Without such proven mechanistic detail, designing novel biologically relevant surfaces, and understanding the potential and limitations of present ones is haphazard at best.

Herein, we address this challenge by studying the biotin-KKKKC:TEGT mixed SAM as a model system and report the first observation of biotin orientations in the charged mixed SAM in response to an applied potential, using in situ SFG spectroscopy. This technique takes advantage of even-order non-linear optical selection rules that precludes signals from isotropic environments or molecular arrangements that possess inversion symmetry.^24^ At interfaces, the symmetry is necessarily broken making even-order processes, such as second-order SFG, intrinsically surface specific.^25^ In a typical SFG experiment, IR and visible laser pulses are overlapped in time and space at an interface to eventually generate a sum-frequency signal. Spectra are recorded as a function of IR frequency; SFG signals are resonantly enhanced when the IR light is exciting vibrational states that are both IR and Raman active. In the realm of SAMs used in electrochemical settings, SFG spectroscopy has been applied to study chemical, orientational, and conformational changes within SAMs at electrified interfaces.^26^–^35^ While the majority of these studies focus on the use of aliphatic and aromatic compounds, herein we use for the first time in situ SFG spectroscopy to investigate changes in molecular orientations in charged biofunctionalized SAMs in response to an applied electrical potential. This is particularly challenging due to the comparably lower density of charged biomolecules on the surface and a resulting greater degree of conformational freedom. Both effects decrease SFG signals, which are depending on density and order.

The SFG characterization was performed with an easy to assemble and purpose built electrochemical cell that allowed recording of SFG spectra while applying an electrical potential. Furthermore, the conformational changes of the biotin-KKKKC:TEGT SAMs observed by SFG at negative and positive potential are compared with densely packed biotin-KKKKC SAMs to understand the importance of the presence of TEGT as a spacer group. SPR data are also discussed to further strengthen our findings. Finally, the reversibility of the conformational changes is verified.

The switching properties of the mixed SAMs were investigated by applying an electrical potential as an external stimulus. SAMs of biotin-KKKKC:TEGT were formed directly onto a 15 nm gold coated side of an equilateral CaF_2_ prism (size 25 mm). The prism was then placed onto a purpose built Teflon electrochemical cell (**Figure** **2**) containing PBS buffer (pH = 7.4). Applied potentials were measured versus a Ag/AgCl reference electrode (FLEXREF, WPI, USA). Reference and counter electrode (Pt wire) were placed about 3–5 mm below the gold coated prism, which was used as the working electrode. Details on the SAM preparation and picosecond SFG setup (EKSPLA, Lithuania) can be found in the Supporting Information.

**Figure 2:**
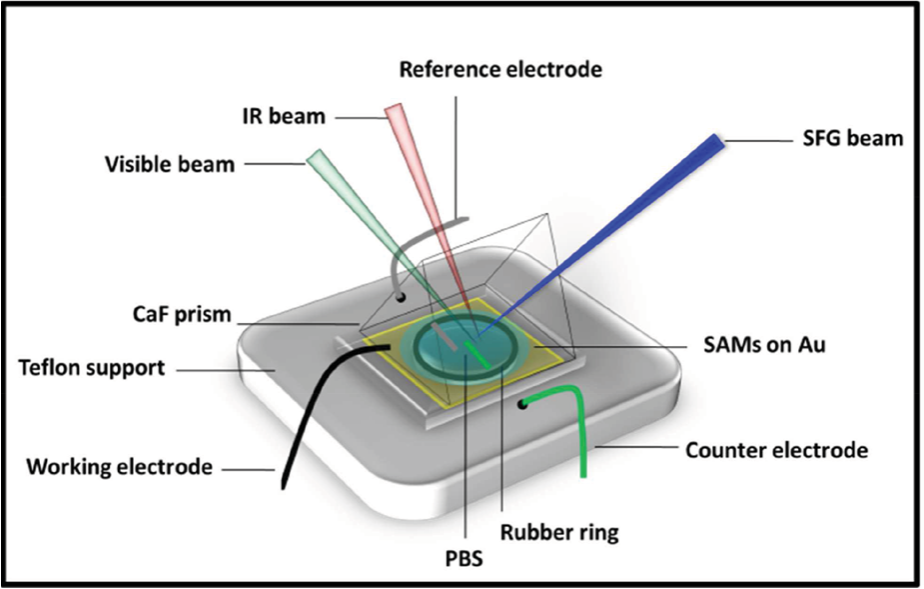
Schematic representation of the spectro-electrochemical cell.

SFG measurements were performed at static potentials. First, a potential of +0.3 V was applied to the biotin-KKKKC:TEGT SAM and the corresponding SFG spectra was recorded. Subsequently, a potential of –0.4 V was applied to the same substrate while the SFG setup was left unchanged. The corresponding spectra (normalized to the IR and visible intensities of the incoming beams) are shown in **Figure** **3** for the region between 3150 and 3350 cm^−1^. While changing the potential from positive to negative values, SFG signals nearly overlap, except for spectral contributions around 3245 cm^−1^. While NH vibrations of the peptide are occurring at frequencies centered above 3280 cm^−1^^36^ and CH vibrations are significantly lower (below 3000 cm^−1^), the peak around 3245 cm^−1^ can be attributed to molecular vibrations within the heterocyclic imidazole moiety of the biotin group.^37^

**Figure 3:**
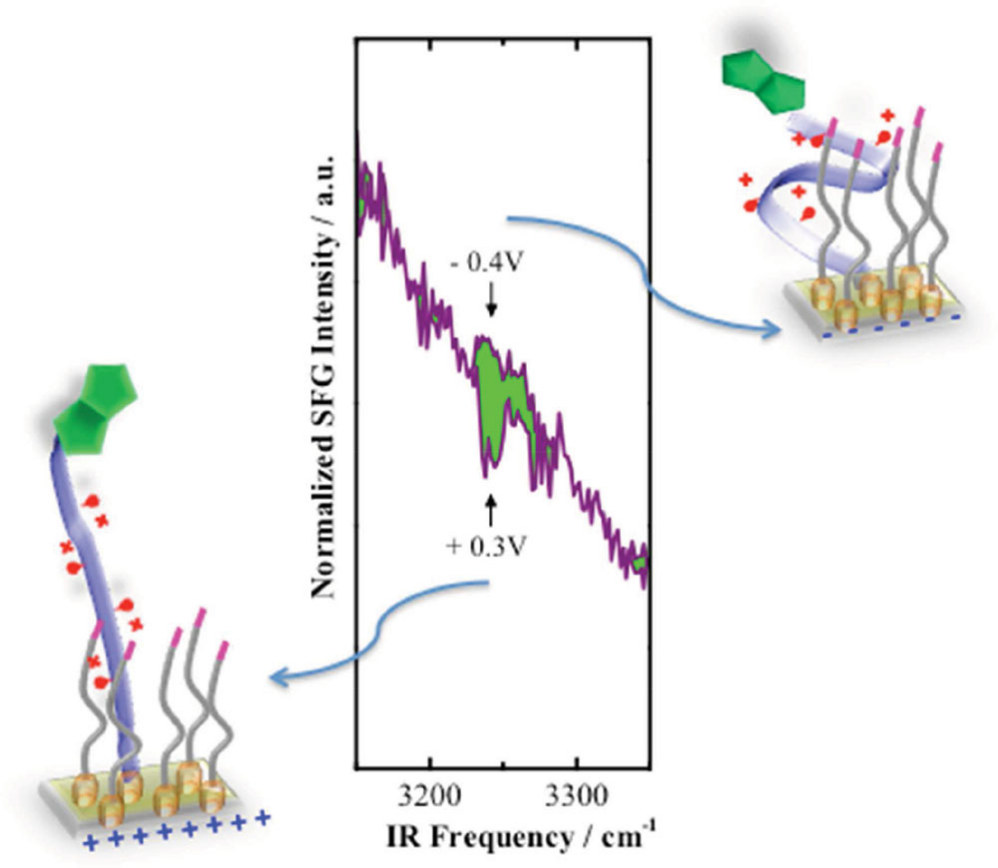
SFG spectra biotin-KKKKC:TEGT at +0.3 V and –0.4 V. Differences between the spectra are marked in green. The illustrations are interpretations of the corresponding molecular arrangement at positive and negative surface potentials.

Besides the narrow band visible in Figure 3, a rather steep incline in the overall spectral shape is observable that is associated to non-resonant signals from electronic transitions within the Au substrate, Fresnel coefficients that change with frequency, and typically broad OH contributions from water.^38^ SFG control studies performed with the single component biotin-KKKKC SAM show that no switching occurs, presumably due to the high level of packing of the oligolysine chains that are constrained in one conformation (Supporting Information). Further evidence of this non-switching behaviour is provided by electrochemical SPR (Supporting information).

An analysis of the relative phase in between the resonant and non-resonant signals can retain information on the orientation of this particular group. A dip in the spectrum is related to a mean orientation of the corresponding transition dipole moment (TDM) away from the substrate (destructive interference), while a peak is indicating an orientation towards the substrate (constructive interference). Applied to the spectra in this present research, the biotin moiety contributing in this spectral region has its mean TDM orientation pointing away from the substrate at positive potential resulting in a dip in the spectrum. In this scenario, the positively charged peptide chain is prone to adopt a conformation that will extend itself away from the substrate due to electrostatic repulsion resulting in an anisotropic upright orientation of the biotin group.

At negative potential, the peptide chains are likely to adopt a collapsed folded conformation due to electrostatic attraction between the negative potential of the surface and the positive charges on the peptide backbone, which appears to have resulted in a disordered biotin group since the SFG signal is no longer visible (isotropic molecular ordering cannot generate SFG signals), Figure 3.

Oftentimes *in situ* SFG spectra of biomolecules at surfaces are rather complex and relatively weak due to the isotropic nature coming with a less tightly packed arrangement of molecules. The mixed biotin-KKKKC:TEGT SAM used in our study has been previously^13^ characterised by X-ray photoelectron spectroscopy (XPS) and an average ratio on the surface of 1:16 ± 4 was observed. It is remarkable that at such a small surface coverage, SFG signals of biomolecules in solution still deliver a significant contribution above the noise ratio. So, our experiments are highlighting the importance of following changes in SFG spectra while changing external parameters such as surface potentials. In this respect, the combination of electrochemistry and SFG, as applied in this study, provides a powerful platform when it comes to in situ spectral analysis utilizing SFG in the context of biointerfaces. Furthermore, the applied potential can be switched back and forth to reproducibly cycle between the two spectral states. Moreover, the observed spectral features might be only slightly above noise level, but the reproducibility strengthens evidence and provides statistical means to an otherwise only singular event. Cycling the external parameter also allows investigating the reversibility of molecular conformations as discussed in the following paragraphs.

**Figure** **4**a shows baseline corrected normalized SFG spectra that have been recorded at +0.3 V, −0.4 V, and back to +0.3 V applied potential. The ability to turn on and off the upwards orientation with the applied potential allows us to monitor the molecular reorientation of the biotin group. The reappearance of biotin peaks at positive potential shows that the biotin group can be reversibly switched from being isotropically oriented at negative potential towards an anisotropic orientation at positive potential.^39^ The corresponding fitted intensities for the spectra shown in Figure 4a can be found in Figure 4b. Positive and negative potentials are clearly separated demonstrating that the identification of 2 states (upright and random orientation) is above noise level. Additionally, mean values at repeating positive potential are within the error of the spectral fit quantifying the reversible nature of the switching process.

**Figure 4:**
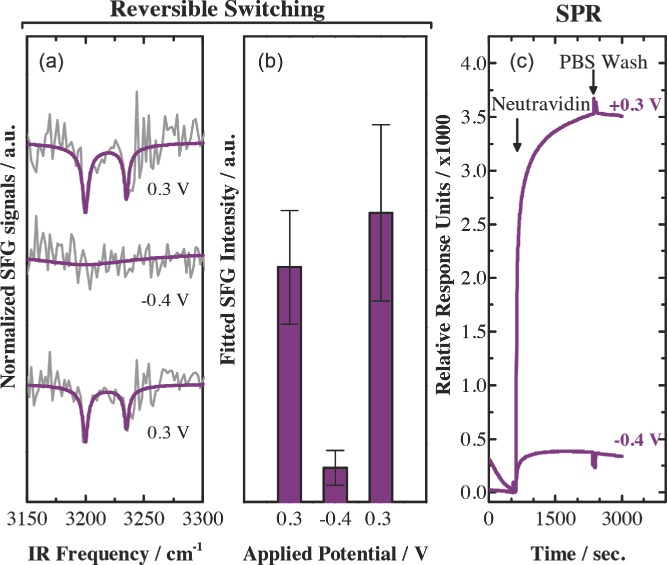
a) Normalized and baseline corrected SFG spectra of the Biotin-KKKKC:TEGT SAM (grey lines) and their corresponding fits (purple lines) for +0.3 V, –0.4V, and returning to +0.3 V. b) Sum of the fitted resonant SFG intensity (represented through amplitude divided by width) at switching surface potentials. c) SPR sensorgram traces showing the binding of Neutravidin (37 μg mL^−1^) to the biotin-KKKKC:TEGT mixed SAMs at a solution ratio under an applied positive (+ 0.3 V) and negative (−0.4 V) potential. After Neutravidin binding for 30 min, the surfaces were washed with PBS for 20 min to remove any nonspecifically adsorbed Neutravidin.

The conformational change of the biotin-KKKKC peptide is further illustrated by SPR measurements of the binding events between the biotin end-group of the biotin-KKKKC:TEGT SAM and the neutrally charged protein Neutravidin at different applied potential (Figure 4c). For biotin-KKKKC:TEGT SAM, the binding process is favoured at +0.3 V when the oligolysine backbones are in an extended conformation and the biotin end-groups are exposed. In contrast, at –0.4 V the interaction between the biotin and the Neutravidin is prevented due to the folded conformation of the backbones which makes the biotin unavailable. On the other hand, when the same experiment is performed on a pure biotin-KKKKC SAM no difference in binding events is observed (see Supporting Information), indicating that despite the different potentials applied no molecular conformational changes are occurring.

Although it is important to study the switching behaviour of SAMs with different experimental detection techniques, no information at the molecular level can be gained by SPR. Furthermore, the reversibility of the switching monitored by SPR implies the use of specific analytes which could lead to several issues such as the occurrence of non-specific binding and the irreversible chemical bonds between the analyte and the ligand. Both these circumstances have an impact on the reversibility performance and therefore on its analysis. However, these difficulties are overcome by using SFG spectroscopy where no extra binding processes are needed (Figures 4a and b).

We have demonstrated that SFG spectroscopy is a highly sensitive tool able to provide an in depth characterisation of the reversibility of electrically switchable biotin-KKKKC:TEGT SAMs. By studying the orientation of the SFG peak's characteristics of the biotin end-group, the determination of the structural orientation under electro-induced switching was ascertained. The switchable process and its reversibility were assessed by repeatedly switching between positive and negative surface potentials. Monolayers of single component biotin-KKKKC SAMs exhibited no conformational change upon the application of an electrical potential, indicating that due to the high level of packing the oligolysine chains are constrained to one extended conformation (anisotropic molecular ordering). On the contrary, when a negative potential of –0.4 V is applied to the mixed biotin-KKKKC:TEGT SAM, a change in conformation occurs owing to the presence of the spacer, TEGT, presumably allowing the folding of the oligolysine backbones (isotropic molecular ordering). Furthermore, the reverse phase of the SFG signal in the region between 3200–3300 cm^−1^ at –0.4 V suggest that the biotin end-group is facing in the opposite direction compared to the initial measurement (+0.3 V). Such information has the potential to positively impact the field of biosensors as well as surface engineering where the direct knowledge of the structure and the geometry of the molecules at interfaces are vitally important.

## Supporting Information

Supporting Information (experimental details of SAM reparation and characterization) is available from the Wiley Online Library or from the author.

As a service to our authors and readers, this journal provides supporting information supplied by the authors. Such materials are peer reviewed and may be re-organized for online delivery, but are not copy-edited or typeset. Technical support issues arising from supporting information (other than missing files) should be addressed to the uthors.
