# Supplementary material for: Direct Observation of Reversible Biomolecule Switching Controlled By Electrical Stimulus
Source: Adv Mater Interfaces. 2014 Apr 4;1(5):1–4. doi: 10.1002/admi.201400026 (PMC4368128; doi:10.1002/admi.201400026)
Supplement: Supplementary file 1 — Supplementary [file admi0001-0001-sd1.pdf]

Copyright WILEY-VCH Verlag GmbH & Co. KGaA, 69469 Weinheim, Germany, 2014.

ADVANCED MATERIALS INTERFACES

Supporting Information

for *Adv. Mater. Interfaces,* DOI: 10.1002/admi.201400026

Direct Observation of Reversible Biomolecule Switching Controlled By Electrical Stimulus

Alice Pranzetti, Matthew Davis, Chun L. Yeung, Jon A. Preece, Patrick Koelsch, and Paula M. Mendes**

Supporting Information

Copyright WILEY-VCH Verlag GmbH & Co. KGaA, 69469 Weinheim, Germany, 2013.

Direct Observation of Reversible Biomolecule Switching Controlled By Electrical Stimulus

Alice Pranzetti, Matthew Davis, Chun L. Yeung, Jon A. Preece, Patrick Koelsch, Paula M. Mendes**

Alice Pranzetti, Dr Chun L. Yeung, Prof. Paula M. Mendes

School of Chemical Engineering, University of Birmingham, Edgbaston, Birmingham, B15 2TT, UK.

Email: p.m.mendes@bham.ac.uk

Prof Jon A Preece

School of Chemistry, University of Birmingham, Edgbaston, Birmingham, B15 2TT, UK.

Matthew Davis and Prof. Patrick Koelsch

National ESCA and Surface Analysis Center for Biomedical Problems, Department of Bioengineering, University of Washington, P.O. Box 351653, Seattle, Washington 98195-1653, USA .

Email: koelsch@uw.edu

1. Chemicals and Materials

Commercially available chemicals and solvents were purchased from Aldrich Chemicals and Fisher Chemicals and were used as received. The oligopeptide biotin-KKKKC was synthetised by Peptide Protein Research Ltd. (Wickham, UK) to > 95% purity and verified by HPLC and mass spectrometry. Neutravidin was purchased from Invitrogen. Triethylene glycol thiol (TEGT) was synthesized as previously described.[1] Phosphate buffered saline (PBS) solution was prepared from a 10× concentrate PBS solution (1.37 M sodium chloride, 0.027 M potassium chloride, and 0.119 M phosphate buffer) from Fisher BioReagents. Polycrystalline gold substrates were purchased from George Albert PVD., Germany and consisted of a 50 nm gold layer deposited onto a glass covered with a thin layer of chromium.

2. SAM Preparation

The gold substrates were cleaned by exposure to UV light for 1 h and immediately rinsed with Ultra High Pure (UHP) H_2O, followed by HPLC grade ethanol for 1 min and UHP. For the preparation of the pure biotin-KKKKC and TEGT SAMs, the clean gold substrates were immersed for 12 h in ethanolic 0.1 mM solution of biotin-KKKKC containing 3% (v/v) $N(CH_2CH_3)_3$ and 0.1 mM solution of TEGT, respectively. For the preparation of the mixed biotin-KKKKC:TEGT SAMs, solutions of the oligopeptide biotin-KKKKC (0.1 mM) and TEGT (0.1 mM) were prepared in HPLC EtOH containing 3% (v/v) $N(CH_2CH_3)_3$, and mixed at the volume ratio of 1:40. Subsequently, the clean gold substrates were immersed in the mixed solution for 12 h to form the mixed SAMs on the gold surfaces. The substrates were rinsed with HPLC EtOH, an ethanolic solution containing 10% (v/v) CH_3COOH, and UHP H_2O and dried under a stream of N_2. Note that the mixed SAMs were deposited in the presence of $N(CH_2CH_3)_3$ to prevent the formation of hydrogen bonds between the NH_2 functional groups of the bound thiolate peptide on Au surface and that of free thiol peptide in the bulk solution.[1]

3. Contact Angle

Contact angles were determined using a home-built contact angle apparatus, equipped with a charged coupled device (CCD) KP-M1E/K camera (Hitachi) that was attached to a personal computer for video capture. The dynamic contact angles were recorded as a micro-syringe was used to quasi-statically add liquid to or remove liquid from the drop. The drop was shown as a live video image on the PC screen and the acquisition rate was 4 frames per second. FTA Video Analysis software v1.96 (First Ten Angstroms) was used for the analysis of the contact angle of a droplet of UHP H_2O at the three-phase intersection. The averages and standard errors of contact angles were determined from five different measurements made for each type of SAM.

4. Ellipsometry

The thickness of the deposited monolayers was determined by spectroscopic ellipsometry. A Jobin-Yvon UVISEL ellipsometer with a xenon light source was used for the measurements. The angle of incidence was fixed at 70°. A wavelength range of 280–820 nm was used. The

DeltaPsi software was employed to determine the thickness values and the calculations were based on a three-phase ambient/SAM/Au model, in which the SAM was assumed to be isotropic and assigned a refractive index of 1.50. The thickness reported is the average and standard error of six measurements taken on each SAM.

5. X-ray photoelectron spectroscopy (XPS)

XPS spectra were obtained on the Scienta ESCA300 instrument based at the Council for the Central Laboratory of the Research Councils (CCLRC) in The National Centre for Electron Spectroscopy and Surface Analysis (NCESS) facility at Daresbury, UK. XPS experiments were carried out using a monochromatic Al K α X-ray source (1486.7 eV) and a take-off angle of 15°. High-resolution scans of N (1s) and S (2p) were recorded using a pass energy of 150 eV at a step size of 0.05 eV. Fitting of XPS peaks was performed using the Avantage V 2.2 processing software. Sensitivity factors used in this study were: N (1s), 1.73; S (2p), 2.08; Au (4f 7/2), 9.58; Au (4f 5/2), 7.54.

6. Characterisation of the biotin-KKKKC, TEGT and biotin-KKKKC:TEGT SAMs

The formation of pure biotin-KKKKC, pure TEGT and mixed biotin-KKKKC:TEGT SAMs was studied by means of contact angle and ellipsometry (Table S1). As expected, the water advancing and receding contact angles for the pure TEGT SAM revealed a hydrophilic monolayer. The pure biotin-KKKKC SAM formed a less hydrophilic surface, with biotin-KKKKC:TEGT SAM exhibiting contact angles in between those observed for pure monolayers of either components. This intermediates contact angles values obtained support the formation of the mixed SAM. Ellipsometry analysis of the three surfaces showed the formation of monolayers after 12 h immersion time with thickness values close to the theoretical measurements (obtained from Chem 3D software). In particular, the thickness values observed for the biotin-KKKKC:TEGT SAMs were closer to those found for the TEGT SAMs revealing an higher concentration of TEGT compared to biotin-KKKKC on the surface. The mixed biotin-KKKKC:TEGT SAM has been previously[1] characterised by X-ray photoelectron spectroscopy (XPS) and an average ratio on the surface of 1:16 ± 4 was observed.

Table S1. *Advancing and receding water contact angles as well as theoretical and experimental ellipsometric thicknesses for the different SAMs: biotin-KKKKC, biotin-KKKKC:TEGT and TEGT formed for 12 h. In the table a) indicates the molecular length obtained from Chem 3D software; b) indicates the SAM thickness obtained from experimental ellipsometry measurements.*

SAM	Contact Angle (º)		Thickness (nm)	
	Advancing	*Receding*	*Theoretical*[a]	*Experimental*[b]
biotin-KKKKC	40 ± 2	35 ± 3	5.1	2.23 ± 0.19
biotin-KKKKC:TEGT 1:40 (open circuit condition)	34 ± 3	32 ± 2	1.8	1.33 ± 0.13
TEGT	30 ± 2	27± 2	1.6	1.17 ± 0.11

Furthermore, Fourier transform infrared reflection-absorption spectroscopy (FT-IRRAS) analysis of the modified gold surfaces were performed and the characteristic peaks for each of the three SAMs are highlighted in Figure S1. All the SAM surfaces presented peaks in the CH region between 2800-3000 cm^{-1}. In particular, three peaks for the biotin-KKKKC SAM and the biotin-KKKKC:TEGT SAM and two peaks for the TEGT SAM were observed. In addition, the biotin-KKKKC SAM displayed a band at ~1675 cm^{-1} which was readily assigned to the amide band of the peptide groups.[2-3] This strong band in the amide region (1600-1700 cm^{-1}) was not found in the biotin-KKKKC:TEGT SAM. The absence of the amide peak in this latter surface is probably due to the scarce concentration of the biotin-KKKKC peptide compared to the backfiller TEGT. Furthermore, it is important to notice that the bands between 3200-3300 cm^{-1} presented in the biotin-KKKKC and biotin-KKKKC:TEGT SAM are shifted compared to the broad hydroxyl band at ~3350 cm^{-1} displayed in the TEGT SAM.[4]

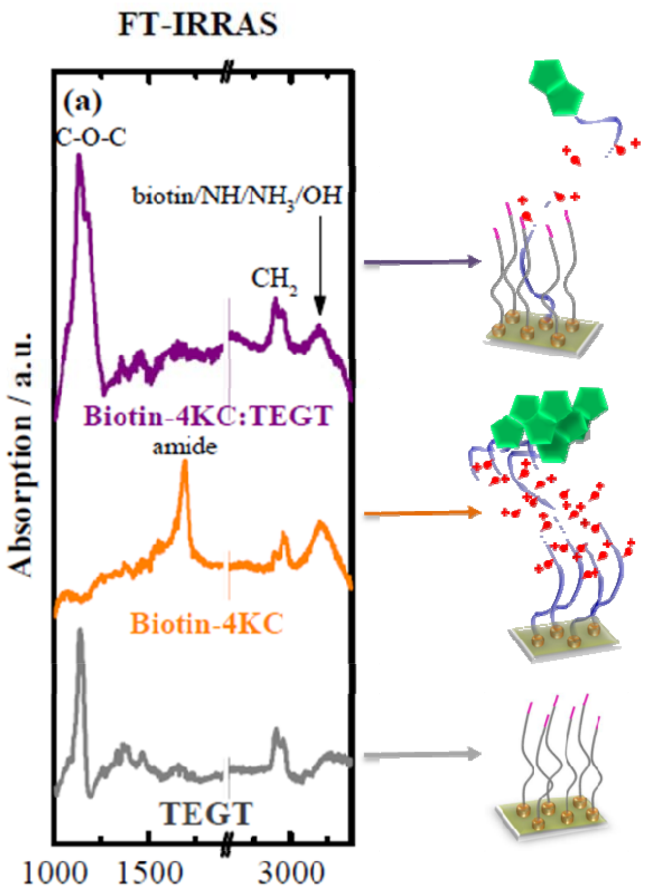

Figure S1. *FT-IRRAS spectra of biotin-KKKKC, biotin-KKKKC:TEGT and TEGT.*

7. Electrochemical Surface Plasmon Resonance (SPR)

SPR switching experiments were performed with a Reichert SR7000DC Dual Channel Spectrometer (Buffalo, NY, USA) at 25°C using a three-electrode electrochemical cell and a Gamry PCI4/G300 potentiostat. The SAMs prepared on Reichert Au sensor chips served as the working electrode, the counter electrode was a Pt wire, and a standard calomel electrode (SCE) was used as the reference electrode. Prior to the Neutravidin binding studies, the sensor chips were equilibrated with degassed PBS, followed by application of either + 0.3 V, − 0.4 V or open circuit conditions for 10 min while passing degassed PBS through the electrochemical cell at a flow rate of 100 μL min^{-1}. While still applying a potential, Neutravidin (500 μ L, 37 μ g mL^{-1}), was injected over the sensor chip surface for 10 s at 1500 μL min^{-1} and then 30 min at 8 μL min^{-1} (the decrease in flow rate from 1500 to 8 μ L min^{-1} ensures that sufficient exposure time is provided for binding to occur between the biotin on the surface and Neutravidin in solution). In order to remove any unbound Neutravidin, the sensor chips were

washed with degassed PBS for 10 s at a flow rate of 1500 μL min^{-1}, followed by 20 min at a flow rate of 100 μL min^{-1} while still applying a potential to the chips.

8. SAM formation on the gold coated CAF_2 prism

CaF_2 prisms were coated on one side with a 15 nm thick Au layer by chemical vapor deposition. All Au-coated prisms were cleaned by exposure to UV light for 20 min and immediately rinsed with UHP H_2O, followed by HPLC grade ethanol for 1 min and UHP. The prisms were then immersed into either a solution of biotin-KKKKC or biotin-KKKKC:TEGT prepared as described in *Section 2-SAM preparation*. In order to avoid the solvent evaporation, the procedure described was carried out in a purpose built chamber. The substrates were rinsed with HPLC EtOH, an ethanolic solution containing 10% (v/v) CH_3COOH, and UHP H_2O and dried under a stream of N_2.

9. Sum Frequency Generation (SFG)

SFG spectra of the biotin-KKKKC SAMs and biotin-KKKKC:TEGT SAMs were acquired under *in situ* and in air conditions in order to monitor the spectral regions previously assessed by FT-IRRAS. SFG signals of molecules on an Au substrate can have two contributions: i) a nonresonant contribution emanating from electronic transitions within the Au layer and ii) resonant ones that are originating from vibrational transitions within molecules at the interface. The slope of the nonresonant Au-signal is depending on changes in Fresnel coefficients with frequency and can be, therefore, often found to be non-zero. Typically, narrowband resonant signals appear as dips or peaks on the nonresonant signal. Figure S2 is showing SFG spectra of a biotin-KKKKC SAM on Au and mixed biotin-KKKKC:TEGT SAM on gold wafers in air and in open circuit (OC) conditions. Both spectra show resonant signals in the CH stretching frequency region between 2800 and 3000 cm^{-1}. In particular, for the biotin-KKKKC SAM the spectrum shows resonances for the CH_2 (2858 cm^{-1}) symmetric stretch mode and for the CH_2 asymmetric stretch modes at 2890 cm^{-1}, while the two peaks at 2946 and 2975 cm^{-1} are assigned to the stretching vibrations of the single CH_2 group pertaining to the ureido bicycle of the biotin molecule end-group.[5] The presence of the additional small and sharp band at ~3020 cm^{-1} could be associated to a Fermi resonance-enhanced overtone coming from the amide II entities[6] as well as the NH_3^+ stretching of the oligolylisine backbone of biotin-KKKKC. However, available literature report these

stretching at higher frequencies (~3300 cm^{-1})[7],[8-9]. The fact that this contribution is appearing as a dip on the nonresonant signal is indicative of an orientation of the transition dipole moment (TDM) away from the surface. Within the mixed SAM, features in these spectral regions are less pronounced and of more difficult interpretation. However, a small peak at ~3220 cm^{-1} could still be observed.

The differences between the SFG spectra of biotin-KKKKC and biotin-KKKKC-TEGT could be mainly imputed to the lower surface density for the latter surface and the higher rate of disorder of the molecules which can arrange in a greater variety of conformations. As a result, the sum of TDM from this more isotropic system is lower in comparison to an ordered system such as the biotin-KKKKC SAM in air. Therefore, as the strength of SFG signals depends on both density and order, the intensity is less pronounced in the case of the mixed SAM. Furthermore, this SAM might not be uniformly mixed throughout the sample area and domains of either biotin-KKKKC or TEGT may be present on the surface.

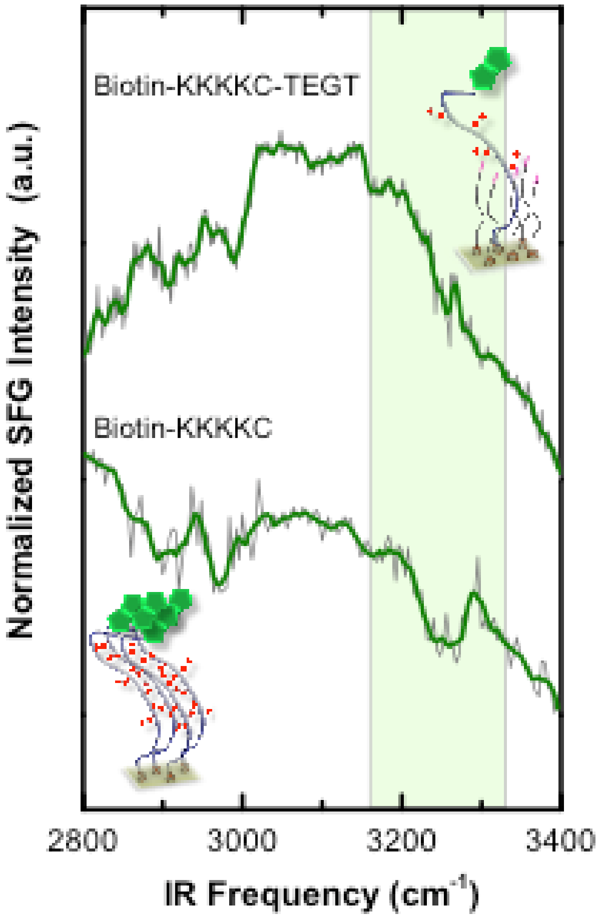

Figure S2. *SFG spectra of biotin-KKKKC and biotin KKKKC:TEGT in air and in OC.*

10. Electrochemical Sum Frequency Generation (SFG)

The electroinduced conformational changes of the biotin-terminated oligolysine peptide SAMs were monitored under *in situ* conditions by SFG spectroscopy. The SFG spectra have been acquired with a picosecond Nd:YAG laser (PL2241, EKSPLA). The beams have a pulse duration of 35 ps at a repetition rate of 50 Hz. The beam diameter at the sample stage was about 2 mm to avoid ablation of the 15 nm Au film on the CaF_2 prism. Tunable IR pulses (about 200 μJ) are overlapped at the sample interface with a beam of visible (532 nm, 200 μJ) light to produce the output SFG light. The substrate films prepared onto one side of an equilateral CaF_2 prism, as described above, were in contact with the sample solution in a Teflon liquid cell. After immersion of the modified CaF_2 prism into the purpose built electrochemical cell, a positive potential of + 0.3 V was applied. The laser beams were then directed through the backside of the prism to probe the substrate-solution interface *in situ* in near-total internal reflection geometry. The visible and IR beams were overlapped at the sample spatially and temporally with incidence angles of 67° and 55°, respectively. All beams (SFG, VIS, and IR) were p-polarized. The potential was applied during all the spectral acquisition (~40 minutes) and was inverted (to -0.4 V) at the end of it. A further SFG spectral acquisition at -0.4 V was then performed (~40 minutes). A maximum of three consecutive SFG runs in presence of a potential applied have been accomplished. The stability of the SAMs during the electrochemical stimulation has previously been proven by us.[1]

11. Control studies performed with biotin-KKKKC SAM

The SFG experiment described above was performed on the biotin-KKKKC SAM in order to prove that switching was only possible due to the space between the peptide moieties provided in the biotin-KKKKC:TEGT SAM. Figure S3a shows the overlapping of the biotin-KKKKC SFG spectra recorded at positive and negative potential.

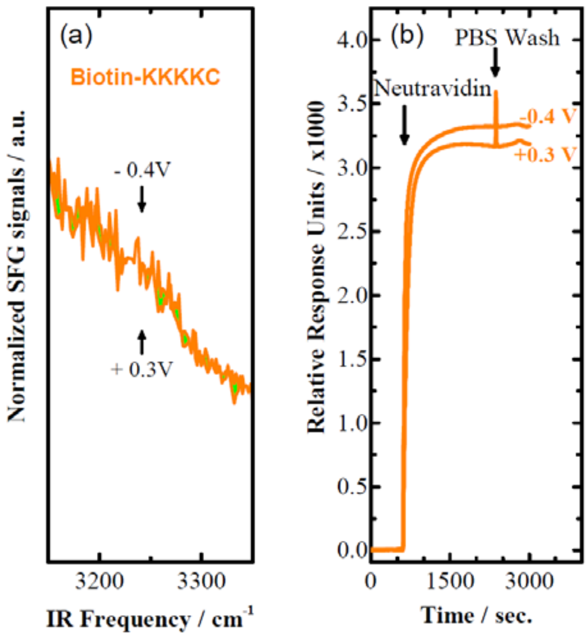

Figure S3. *a) SFG spectra of biotin* KKKKC *at + 0.3 V and – 0.4 V. Differences between the spectra are marked in green. b) SPR sensorgram traces showing the binding of Neutravidin (37 µg mL^{-1}) to the biotin-KKKKC SAMs under an applied positive (+ 0.3 V) and negative (− 0.4 V) potential. After Neutravidin binding for 30 min, the surfaces were washed with PBS for 20 min to remove any nonspecifically adsorbed Neutravidin.*

Both the SFG spectra and the SPR results show that there are no significant changes in the film between positive and negative potential applied. Isotropic arrangement largely remains for the biotin-KKKKC SAM due to steric hindrance, therefore no detectable reorientations for the biotin occurs as opposed to the biotin-KKKKC:TEGT film, in which the peptides have sufficiently space to reconfigure towards a random orientation. In particular, the SFG spectra at positive and negative potential are almost completely overlapping but the peak at 3220 cm^{-1} is not present suggesting a higher disorder rate of the surfactant molecules compared to the same surface analysed in air (Figure S2). Figure S3b shows the Relative Response Units (RRU) found by electrochemical SPR analysis for biotin-KKKKC SAMs. The difference between the sensorgrams obtained at the two potentials is less than 100 RRU and the affinity of the biotin group for the Neutravidin is still high in both cases, indicating that the interaction biotin-NeutrAvidin is independent from the alignment of the biotin groups. Figure S4 shows the proposed model for the different molecular conformations in OC (in air) and under electrochemical stimulation (in solution).

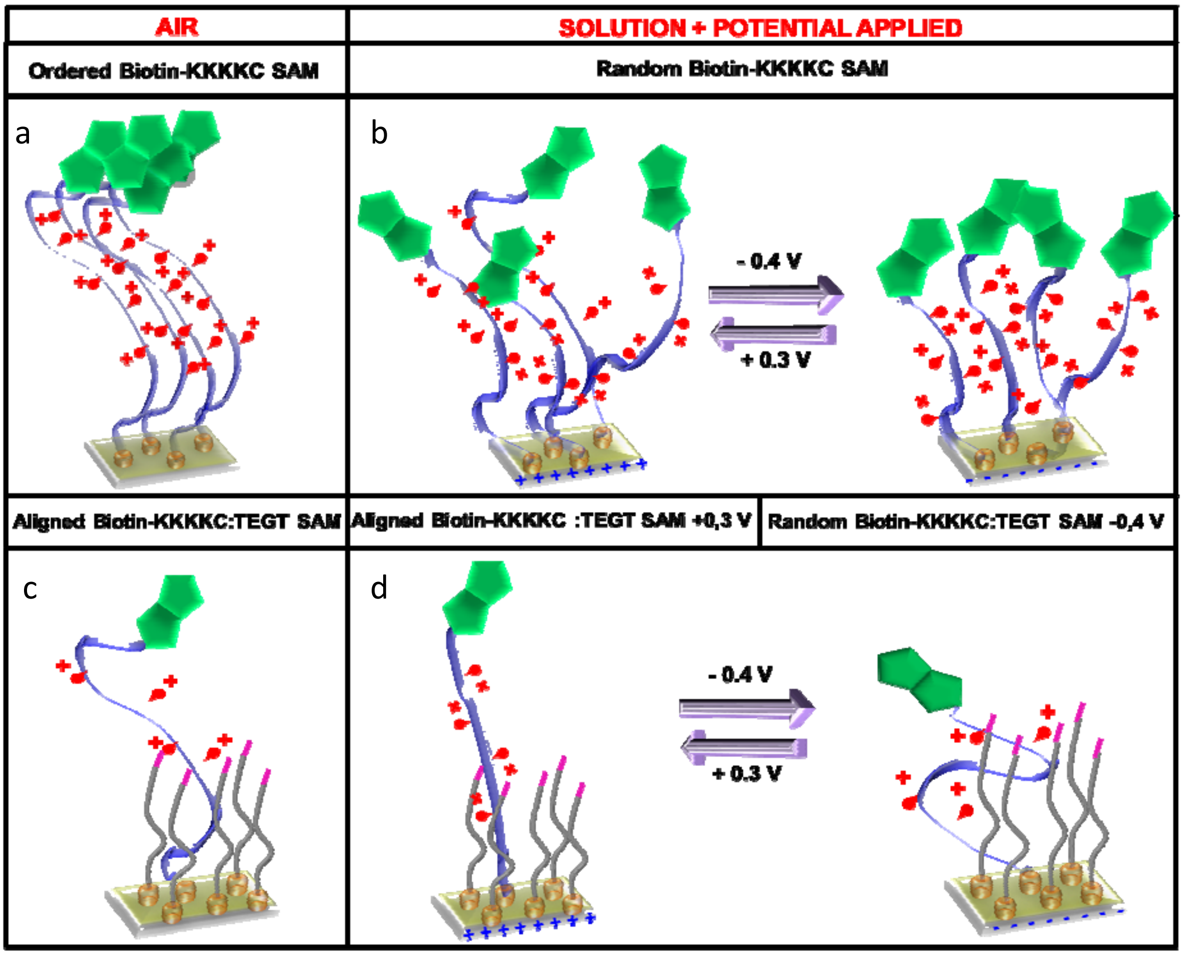

Figure S4. *Model showing the different orientation of the Biotin-KKKKC when a) Densely packed and aligned in air at OC; b) Misaligned in solution and under electrochemical stimulation; c) aligned but weakly concentrated in air at OC and d) Aligned at positively potential and randomly oriented at negative potential in solution.*

[1] C. L. Yeung, P. Iqbal, M. Allan, M. Lashkor, J. A. Preece, P. M. Mendes, *Adv. Funct. Mater.* **2010**, *20*, 2657–2663

[2] R. S. Clegg, J. E. Hutchison, *Langmuir* **1996**, *12*, 5239-5243.

[3] R. Valiokas, S. Svedhem, S. C. T. Svensson, B. Liedberg, *Langmuir* **1999**, *15*, 3390-3394.

[4] S. Jo, P. S. Engel, A. G. Mikos, *Polymer* **2000**, *41*, 7595-7604.

[5] G. Tourillon, L. Dreesen, C. Volcke, Y. Sartenaer, P. Thiry, A. Peremans, *Journal of Materials Science* **2009**, *44*, 6805-6810.

[6] Lin-Vien D, Colthup NB, Fateley WG, G. JG, *The handbook of infrared and raman characteristic frequencies of organic molecules.*, Academic Press,, San Diego, CA, **1991**.

[7] O. Mermut, D. C. Phillips, Roger L. York, K. R. McCrea, R. S. Ward, G. A. Somorjai, *J. Am. Chem. Soc.* **2006** *128*, 3598-3607

[8] T. Weidner, N. F. Breen, G. P. Drobny, D. G. Castner, *Journal of Physical Chemistry B* **2009**, *113*, 15423-15426.
[9] B. G. Nidumolu, M. C. Urbina, J. Hormes, C. Kumar, W. T. Monroe, *Biotechnology Progress* **2006**, *22*, 91-95.
